# The Between-Competition Running Demands of Elite Hurling Match-Play

**DOI:** 10.3390/sports9110145

**Published:** 2021-10-22

**Authors:** Brendan Egan, Damien Young, Kieran Collins, Shane Malone, Giuseppe Coratella

**Affiliations:** 1 Department of Sport and Early Childhood Studies, Technological University of the Shannon: Midlands Midwest, Thurles Campus, Thurles, E41 PC92 Tipperary, Ireland; brendanegan11@gmail.com; 2 Department of Science, Gaelic Sport Research Centre, Technological University Dublin, Tallaght, D24 FKT9 Dublin, Ireland; kieran.collins@tudublin.ie (K.C.); shane.malone@mymail.ittdublin.ie (S.M.); 3 Research Institute for Sport and Exercise Sciences, Liverpool John Moores University, Liverpool L3 3AF, UK; 4 Department of Biomedical Sciences for Health, University of Milan, 20133 Milan, Italy; giuseppe.coratella@unimi.it

**Keywords:** GPS, sprints, high-speed distance, team sports, performance analysis, championship

## Abstract

The current study aimed to investigate the differences in running demands between the National Hurling League (NHL) and the Championship, and within playing positions. GPS (10 Hz, STATSports Apex GNSS) were used to analyse the running demands during 34 games (2017–2020 seasons) of the Championship and the NHL. The running demands (total-, relative-, high-speed- [>17 km·h^−1^] and sprint [≥22 km·h^−1^] distance, number and length of sprints, and peak speed) were compared between competitions. Greater total- [ES = 0.32], relative- [ES = 0.26], and sprint-distance [ES = 0.41], and number of sprints [ES = 1.29] were completed in the Championship. The high-speed distance was similar between competitions. Half-backs and half-forwards covered greater total- (ES = 0.91 and 0.21, respectively), relative- (ES = 1.14 and 0.68, respectively), high-speed- (ES = 0.69 and 0.44, respectively), and sprint-distance (ES = 0.50 and 1.26, respectively), number of sprints (ES = 2.66 and 1.73, respectively), and peak speed (ES = 1.09 and 1.32, respectively) in the Championship. There was no difference (*p* < 0.05) in the sprint distance covered between positions in the Championship. The results showed that the Championship is more physically demanding. The findings present key implications for the transition between competitions.

## 1. Introduction

Hurling is a stick and ball invasion field sport and it is one of the national sports played in Ireland [1]. It is a physically demanding intermittent high-intensity sport [2]. Players have typically covered distances of ~850 m, and ~340 m at high-speed running and sprinting speeds, respectively and performed ~24 sprints during senior games [1]. It is also a highly skilled sport, consisting of catching, striking, and tackling [1,3]. Two teams of 15 players aim to outscore each other by striking the ball through the opposing team’s goalposts [1]. Each team is divided into five positional lines; full back, half back, midfield, half forward and full forward. The best players at the sub-elite level (club) are chosen to compete for their senior county team in the Provincial and All-Ireland Championship [3]. The elite senior competitive season is divided into two competitions, the National Hurling League (League) (January to April) and the Championship (May to August) [4]. Each competition consists of the top twelve elite teams in Ireland. Teams play a maximum of nine and seven games in the League (six league rounds, quarter-final, semi-final and final) and Championship (four league rounds, Provincial final or quarter-final, semi-final and final), respectively. The League is often used as a pre-season competition, whereas the Championship is considered the most important, with the All-Ireland final viewed as one of the biggest sporting events of the year in Ireland [5]. The match-play running demands of hurlers have previously been reported [1,6,7,8]. However, no research has differentiated between the two elite senior hurling competitions. Providing this information would help coaches periodise their training programs to meet the demands of both the League and Championship competitions.

Global positioning system technologies (GPS) have become a key device used by sport scientists and coaches to understand the physical demands of matches and to quantify the physical load performed by players in training [9]. These GPS technologies have been shown to provide valid and reliable measures of running performance in team sports [10,11]. Several studies have reported the match-play running performances in soccer [12,13,14,15], rugby [16,17,18], Gaelic football [19,20], field hockey [21,22], and hurling [1,6,7,8,23] using GPS. Specifically, the total distance, distance per minute, distance covered at high-speed running (HSR) and sprinting, the number and length of sprints, and the peak speeds have been quantified [1,6,7,8]. When the physical demands of the sport have been established, coaches can design training content and seasonal plans to prepare the players to withstand the high-intensity exercise demands of competition [24,25]. Recently, GPS has been utilized to quantify the match-play running demands of elite senior hurling [1,6,7,8,23]. Previous research in hurling have investigated the running demands for the full-game, between halves and positions [1,6,7,8]. Senior hurlers (~112 m·min^−1^) covered similar relative distance compared with Gaelic football (~116 m·min^−1^) [19] and soccer (~113 m·min^−1^) [12], but larger than that found in rugby (~85 m·min^−1^) [16]. A drop-off in the total distance (~3950 m vs. ~3858 m), relative distance (~113 vs. ~110 m·min^−1^), HSR (~448 m vs. ~403 m), and total sprint distance (~178 m vs. ~162 m) was found in the second half in senior hurling games [1]. Furthermore, differences were found between-positions, the half-backs, midfielders, and half-forwards covered greater TD and HSR compared to full-backs and full-forwards [1,2,9]. However, between-position differences were less obvious in the sprint demands (5). To date, research in hurling have included data from both the League and Championship within the same data collection [1,6,7,8]. Given the League is played at the start of the year while some teams are in the middle of pre-season training, the running demands may be lower than performed later in the year during the Championship. Seasonal changes in the running demands have been reported in Gaelic football [19,26], Australian football (AFL) [27] and soccer [24,28]. A greater total distance and high-speed distance were covered in the final stages of the Gaelic football Championship (August and September) compared to the months during which the National Football League (January to April) was played [26]. Reporting the mean data from the League and Championship together may underestimate the intensity of the hurling Championship games. Coaches who design training based on the existing data may be underpreparing players for the possible greater running demands of Championship. Therefore, the current study aims to investigate the differences in running performances between the League and the Championship over the full game and within playing positions. It is hypothesised that there would be greater running demands in the full game and within positions during Championship games compared to the League.

## 2. Materials and Methods

### 2.1. Experimental Approach to the Problem

The current observational study was designed to examine the between-competition running demands of elite hurling match-play. All players in the current study were competing at the highest level (Provincial and All-Ireland Senior Championship) and were selected, as they were members of the county’s squad that season (2017–2020). Players were divided into five playing positions: full backs, half backs, midfielders, half forwards, and full forwards. Data were only included if they participated in the full match (70-min). All games (*n* = 34) took place between 14.00 and 21.00 h. GPS was used to determine specific running performance variables.

### 2.2. Subjects

Fifty (*n* = 50) elite senior male hurlers with a mean age, height and bodyweight of 27.2 ± 5.3 years, 182.4 ± 6.2 cm, and 87.3 ± 6.2 kg, respectively, volunteered to participate in this study. The elite team’s coaches selected players to be part of the elite squad based on their physical, technical, and tactical ability [1]. The team’s medical team declared players fit to participate in the games. Therefore, only the players who were free from injury and illness were eligible to partake in this study. Those who were injured and substituted during the game, their data were removed from the study. Before data collection, participants completed at least a 3-month pre-season training phase consisting of both physical conditioning and skills training. A typical week during the League consisted of two to three organised pitch training sessions and two gym sessions, with three organised pitch training sessions and one to two gym sessions during the Championship. Ethical approval was granted by Limerick Institute of Technology Ethical Committee who approved all procedures. The study was conducted according to the Declaration of Helsinki (1975) for studies involving human subjects.

### 2.3. Procedures

The match-play running demands were recorded using 10-Hz GPS microtechnology with a 100-Hz triaxial accelerometer (STATSports, Apex GNSS, Northern Ireland: firmware 4.14 RCO) [1,15]. The validity and reliability of these GPS units for measuring distance and peak speed in team sports has been previously established [10]. The distance bias in the 400 m trial, 128.5 m circuit, and 20 m trial was 1.05 ± 0.87%, 2.3 ± 1.1%, and 1.11 ± 0.99%, respectively. Peak speed measured by the GPS was 26.5 ± 2.3 km·h^−1^, and a radar gun was 26.3 ± 2.4 km·h^−1^. The ICC between Apex GNSS 10 Hz unit and the radar gun was nearly perfect (*p* < 0.001, ICC [90% CI] = 0.96 [0.92; 0.98]). The major finding of this study was that GPS did not underestimate the criterion high-speed distance during a 400-m trial, 128.5 m circuit, and 20 m trial, as well as peak speed [10]. During data collection, the range of satellites was 20 ± 2 and the horizontal dilution of precision was 1.4 ± 2.0. Players were assigned their own unit for the duration of the data collection. Players were required to complete the full game for their data to be included. The players were classified according to their playing position in each match, resulting in the following number of players per position: full backs: *n* = 11, half backs: *n* = 10, midfielders: *n* = 8, half forwards: *n* = 11, full forwards: *n* = 10. GPS units were placed in a pouch in a customised sports vest supplied by the manufacturer, which was worn under the playing jersey [23]. The GPS units were activated before each participant entered the field, allowing for a minimum period of 15 min for initialization and satellite lock [29]. The participants were familiarized with the GPS microtechnology during team training sessions prior to the collection of data. The GPS data were downloaded to a computer using the STATSports, Apex software (firmware: 4.14 RCO) after each game and each data set was labelled with a playing position. Using time stamps, the data were trimmed to only include the actual game-play data, which was then exported to a Microsoft Excel spreadsheet (Microsoft, Redmond, WA, USA) for further analysis.

Data collected from the GPS units included total distance (m), and relative distance (m·min^−1^). The distance covered (m) was further separated into the following zones, high-speed distance (≥17 km·h^−1^), and sprinting (≥22 km·h^−1^) [6]. The number of sprints (n), mean length of sprint (m), and peak speed (km·h^−1^) were also recorded. A sprint was defined by running ≥22 km·h^−1^ for at least 1 s [6].

### 2.4. Statistical Analysis

All statistical analysis was performed using SPSS for Windows (Version 22; SPSS Inc., Chicago, IL, USA). Descriptive analysis and assumptions of normality were verified before parametric statistical analysis was used. Using the Kolmogorov–Smirnov test, the normality of the data distribution was checked, and all dependent parameters were normally distributed (*p* > 0.05). The analysis was performed using a 2-way (position x competition) mixed design (analysis of variance). When an interaction occurred, a Bonferroni post hoc correction was used to detect differences between competition (two levels: League and Championship) and between positions (five levels: full backs, half backs, midfielders, half forwards, and full forwards). The dependent variables across the range of analysis were total distance, relative distance, high-speed distance, sprint distance, the number of sprints (*n*), mean length of sprint, and peak speed with competition levels and playing positions as independent factors. Data are presented as mean ± SD and 95% confidence intervals (CI). Standardized effect size (ES) was calculated with <0.20, 0.20–0.59, 0.60–1.19, 1.20–1.99, and ≥2.00 and interpreted as the following: trivial, small, moderate, large, and very large differences, respectively, as recommended by Hopkins [30]. 

## 3. Results

The total distance (*p* = 0.572 and *p* = 0.293, respectively), relative distance (*p* = 0.576 and *p* = 0.231, respectively), high-speed distance (*p* = 0.255 and *p* = 0.138, respectively), sprint distance (*p* = 0.405 and *p* = 0.345, respectively), the number of sprints (*p* = 0.430 and *p* = 0.269, respectively), mean length of sprint (*p* = 0.626 and *p* = 0.235, respectively), and peak speed (*p* = 0.204 and *p* = 0.035, respectively) for the NHL and Championship were normality distributed. The homogeneity for the NHL and Championship for total distance (*p* = 0.576 and *p* = 0.366, respectively), relative distance (*p* = 0.773 and *p* = 0.129, respectively), high-speed distance (*p* = 0.404 and *p* = 0.315, respectively), sprint distance (*p* = 0.137 and *p* = 0.641, respectively), the number of sprints (*p* = 0.180 and *p* = 0.348, respectively), mean length of sprint (*p* = 0.128 and *p* = 0.971, respectively), and peak speed (*p* = 0.130 and *p* = 0.516, respectively) were non-significant (*p* > 005).

The total game descriptive statistics for total distance, relative distance, high-speed distance, sprint distance, the number of sprints, the mean length of sprint, and peak speed for the Championship and League are presented in Table 1.

The total game descriptive statistics for total distance, relative distance, high-speed distance, sprint distance, the number of sprints, the mean length of sprint, and peak speed per position for the League and Championship are presented in Table 2. Distinct between-competition differences were found on a positional level. Senior hurlers completed a greater (*p* > 0.05) number of sprints in the Championship compared to the League across all positions. No difference (*p* > 0.05) between-competitions were observed in total distance, relative distance, or sprint distance for midfield players.

During the Championship, full backs and full forwards covered less total distance compared to half backs (ES = −5.27 and ES = −5.31, respectively), midfielders (ES = −4.50 and ES = −4.57, respectively), and half forwards (ES = −2.20 and ES = −2.31, respectively). Half forwards covered less total distance compared to half backs (ES = −1.61) and midfielders (ES = −1.46). The results showed that full backs and full forwards covered less relative distance compared to half backs (ES = −3.30 and ES = −3.71, respectively), midfielders (ES = −2.73 and ES = −3.10, respectively), and half forwards (ES = −1.33 and ES = −1.66, respectively). Half forwards covered less relative distance compared to half backs (ES = −1.65) and midfielders (ES = −1.24).

**Figure 1:**
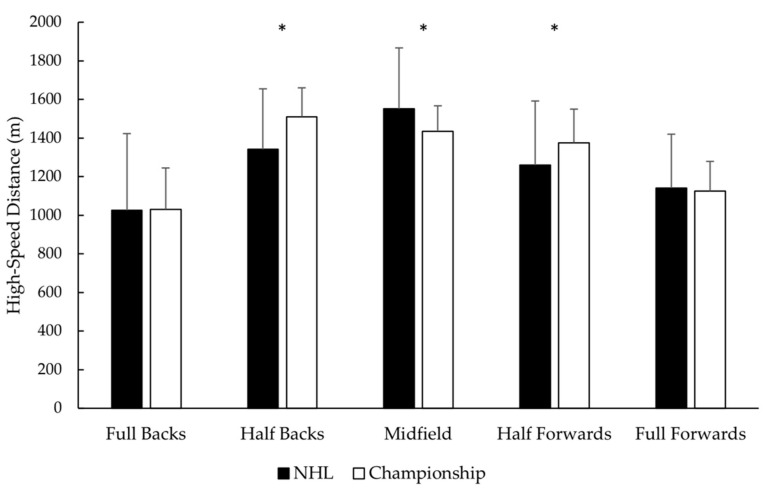
The high-speed distance for the National Hurling League and Championship per position. Data are presented as mean ± SD. NHL = National Hurling League. * Different (*p* < 0.05) from Championship.

Full backs and full forwards covered a lower high-speed distance in comparison to half backs (ES = −2.59 and ES = −2.53, respectively), midfielders (ES = −2.27 and ES = −2.16, respectively), and half forwards (ES = −1.76 and ES = −1.52, respectively) (Figure 1). There was no difference (*p* > 0.05) between positions for the total sprint distance covered during Championship games. Half backs completed a greater number of sprints compared to half forwards (ES = 1.06) and full forwards (ES = 1.73), while full backs and midfielders showed no difference (*p* > 0.05) between positions. Full forwards covered a greater length of sprint compared to full backs (ES = 1.00), half backs (ES = 0.78), and midfielders (ES = 1.00). There was no difference (*p* > 0.05) between half forwards and any other position for the length of sprint covered during Championship games. Full forwards reached a higher peak speed compared to half backs (ES = 0.89) and midfielders (ES = 1.18), while there was no difference observed (*p* > 0.05) between any other position during Championship games.

In the League, full backs and full forwards covered a lower total distance compared to half backs (ES = −1.77 and ES = −2.22, respectively), midfielders (ES = −2.65 and ES = −3.11, respectively), and half forwards (ES = −1.43 and ES = −1.91, respectively). Half forwards covered less total distance than midfielders (ES = −1.10) in the League. The relative distance covered by full backs and full forwards was lower compared to half backs (ES = −1.00 and ES = −0.96, respectively) and midfielders (ES = −1.85 and ES = −1.72, respectively), with half forwards also covering less than midfielders (ES = −1.41). Full backs covered a lower high-speed distance compared to half backs (ES = −0.88) and midfielders (ES = −1.46). Full forwards covered a lower high-speed distance compared to midfielders (ES = −1.37), yet a greater total sprint distance compared to full backs (ES = 0.78). There was no difference (*p* > 0.05) between full backs, half backs, midfielders, and half forwards for the total sprint distance covered during League games. A similar (*p* > 0.05) number of sprints and length of sprint was performed by all positions. Full forwards reached a higher peak speed compared to full backs (ES = 0.77) in League games. There was no other difference (*p* > 0.05) between positions for peak speed.

## 4. Discussion

The purpose of the current study was to describe the between-competition and positional differences in the running demands in elite senior hurling. As hypothesized, greater running demands were observed in the full game and within positions during the Championship compared to the League. The main findings showed that there was greater (small-to-large) total distance, relative distance, sprint distance, number of sprints, and peak speeds in the Championship compared to the League. Distinct positional differences were observed in both Championship and League games, with half backs and midfielders undertaking the greatest work-rate in Championship and League, respectively. To the best of the authors’ knowledge, the current study was the first to examine the running demands of elite senior hurlers between the Championship and League.

There was a greater total distance covered in the Championship compared to the League. The total distance covered previously reported in elite senior hurling [1] was similar to the League results but lower than covered during the Championship. As previous studies have combined the two competitions, this combination of data may have underreported the total distance covered in the Championship [1,6,7,8]. The current results showed that the total distance covered in the Championship is closely aligned to that previously reported in elite Gaelic football [19]. As the League is played towards the end of the pre-season, players may be still developing their fitness capacity compared to months later when they play in the Championship. In addition, teams have limited opportunities to play matches before the League, whereas players may have benefitted from playing the League games during Championship. Therefore, an increase in players’ fitness levels and a greater number of competitive matches completed may have facilitated higher total distance being performed in the Championship. Seasonal increases in running performance have been reported in Gaelic football [26], soccer [28], and AFL [27]. The current results showed similar findings, as the total sprint distance, the number of sprints, and peak speed was greater in Championship (May to September) games compared to the League (January to April). The total sprint distance in both Championship and League is similar to previously reported in hurling [1]. However, the number of sprints completed in the Championship is higher than previous findings in elite senior hurling [1,6]. As the Championship is played in the Summer months (May to September) with warmer weather, the playing surface is firmer compared to the softer surfaces during the wet weather in the League (Winter/Spring). It can be hypothesised that the harder playing surfaces in the Championship may have favoured the number of times that the athletes could reach the sprint threshold, as previously reported in soccer [31,32]. A shorter mean length of sprint and a higher peak speed were observed in the Championship compared to the League. The current study did not analyse the technical skills performed between competitions which may have influenced the running demands. By the time the Championship is played, seven months of technical skills practice would have been completed. This skill refinement may allow players to gain and release possession more efficiently compared to the League. Therefore, the players may release the ball quicker and decelerate from the sprint threshold, which may explain the shorter mean length of sprint in the Championship. The mean length of sprint and peak speeds in the current study are in-line with previous hurling research [1,6]. However, previous research combined both the League and Championship data [1,6]. Overall, the current results highlight the increased running demands found in the Championship, which agree with previous findings in other team sports where seasonal changes were reported [26,27,28]. The use of hurling-specific small-sided games during the league could help players prepare for the higher running demands in competition [33].

Interestingly, there was no difference in the high-speed distance covered between the Championship and the League. The high-speed distance in the current study was similar to previous research in hurling [6]. In contrast, the high-speed distance in Gaelic football increased as the season progressed (August and September) [26]. The authors suggested that the quality of the opposition increased the need for high-speed running [26]. The differences in how players transition between defence and attack in hurling compared to Gaelic football may explain why hurlers complete similar high-speed distances during the Championship and League. In Gaelic football, teams emphasize holding possession while they carry the ball from defence to attack. As the quality of the opposition increases later in the season, the additional high-speed distance may be required to carry the ball past their direct opponent. In contrast, hurlers have the option to strike the ball longer distances (~90 m) from defence to attack. Therefore, the players’ may not be required to carry the ball past the opponents as often as in Gaelic football.

The current study is the first to investigate the positional differences between competitions in hurling. As previously reported in senior hurling [1], half backs, midfielders, and half forwards covered a greater total- and high-speed-distance than full backs and full forwards in both Championship and League. In addition, half backs and midfielders covered a greater total distance than half forwards in the League. Full backs covered a lower high-speed distance in the League than half backs, midfielders, and half forwards with full forwards also covering lower high-speed distances than midfielders. The differences in the positional playing roles and locations on the pitch may explain the greater running demands of the middle three positions. Full backs and full forwards are located close to the goals and the primary roles are to prevent scores and to score, respectively [7]. In contrast, half backs, midfielders, and half forwards are located around the middle third of the field and their role is to link the defence and attack. As a result, these middle three positions have more space to reach the high-speed threshold compared to full backs and full forwards. The current findings support previous research in hurling that found an increased work rate around the middle third of the pitch, in particular, the half back and midfield positions [1].

There was no difference between positions for the total sprint distance covered in the Championship. However, full forwards covered a greater sprint distance than full backs in the League. Teams’ tactical style of play in the League may be different than in the Championship. Full forwards may move further out the field with the aim of gaining more possession of the ball and may emphasize scoring points in the League, while in the Championship full forwards may position themselves closer to the goals with a focus on creating goal-scoring opportunities. There was no difference in the number of sprints between-positions in the League, similar to previous research in senior hurling [1,6]. However, in the Championship, half backs completed more sprints than full backs, half forwards, and full forwards. The role of the half backs has evolved recently. While their team is in possession, the half backs are encouraged to sprint forward to support the forwards while in attack. In addition, this supports the increases in running performance seen in Gaelic football [26], soccer [28], and AFL [27] as the season progresses. The results suggest that all positions may complete similar sprint practices during the League, whereas to prepare for the Championship, players in the middle three positions should be conditioned to perform a greater number of sprints. Full forwards completed a longer mean length of sprint than full backs, half backs, and midfielders in the Championship. However, there was no difference in the mean length of sprint in the League. Even though full forwards performed a longer mean length of sprint in the Championship, this accounted for an ~2.5 m in practical terms.

Between-competition differences were also observed within positions in the current study. Full backs, half backs, half forwards, and full forwards covered greater total distance in the Championship compared to the League. Half backs and half forwards were the only two positions to complete more relative and high-speed distances in the Championship than the League. The 3-month off-season period where teams are not allowed to train collectively [26] may have affected the players’ fitness levels before the League, as they return to pre-season training in a less trained state [34]. Conversely, due to the preceding block of training in the run-to the Championship, players may be in a more trained state, resulting in the increased running performances seen in the current study. There were no differences between competitions in the total- and relative-distance for the midfield players. This highlights a greater work-rate required from the midfield players [1], and suggests that they play an important role in linking defence to attack throughout the season no matter the competition. The current findings showed an increase in the full backs’, half backs’, and half forwards’ total sprint distance in the Championship compared to the League. Overall, there are differences in the running demands between competitions. With the introduction of a standardised GAA fixtures calendar, coaches can use the current results to design and implement a periodised conditioning plan to assist players in completing the higher demands of the Championship.

This study comes with some acknowledged limitations. The current study did not measure the players’ fitness levels before the League or the Championship. With teams having a shorter block of collective training completed prior to the League compared to the Championship, the players’ may have been in a less trained state [34]. This may result in decreased running performances in the League compared to the Championship. Future research should assess the players’ fitness levels prior to the beginning of both competitions and investigate the association between the players’ fitness levels and their running demands. The current study analysed the running demands based on the overall duration of the League and Championship games. Previous research has shown that the ball is only in-play for 40% of the overall match duration (League and Championship games combined) [8]. There may have been differences in the ball-in-play between competitions which may have influenced the current results. Future studies should analyse the running demands of ball-in-play and ball-out-of-play between competitions. No attempt was made to analyse the differences in the technical skill performances between competitions. As players would have performed ~5 months of technical skill practice prior to the Championship, their skills may have been more efficient compared to during the League. Future studies should investigate the impact that technical skill performances have on the players’ running demands. Finally, the current study focused on the physical demands at the end of the game and not the process that generated the result. Future studies should investigate the connection between the playing model and the match running performance.

Some important practical applications appear considering the current findings. The results from this study may aid the design of hurling-specific conditioning to help prepare players for the higher running demands of Championship. Specifically, players should be conditioned to cover greater total- and sprint-distance, perform a greater number of sprints, and reach higher peak speeds to prepare them for the Championship. Coaches should set up activities with longer distances or perform additional repetitions and sets to increase the volume of the total- and sprint distance covered. When preparing players for the high-speed demands of Championship, coaches should allow sufficient rest between activities so that players can reach the required high speeds. The half backs and half forwards’ total-, high-speed-, and sprint-distance, number of sprints, and peak speeds are greater in the Championship compared to the League. Players in these positions should be prepared for the higher demands of the Championship. In addition, midfielders performed a greater high-speed distance during the League. Coaches should be aware of the midfielders’ ability to perform these high-speed demands early in the season. Midfielders’ may be substituted during the League if they are not prepared to meet these early season demands. As the running volume and intensity increases in the Championship, coaches may consider preparing the players to perform these skills at a higher intensity.

## 5. Conclusions

The current study was the first to describe the between-competition and positional differences in the running demands in elite senior hurling. The findings showed that there was greater (small-to-large) total distance, relative distance, sprint distance, number of sprints, and peak speeds in the Championship compared to the League. Half-backs and half-forwards covered greater total-, relative-, high-speed-, and sprint-distance, number of sprints, and peak speed in the Championship. However, there was no difference (*p* < 0.05) in the sprint distance covered between positions in the Championship. The results from this study may aid the design of hurling-specific conditioning to help prepare players for the higher running demands of Championship.

## Figures and Tables

**Table 1:** The total Championship and National Hurling League total running values are reported. Data are presented as mean ± SD, mean difference (95% CI) and effect size.

Performance Variable	League	Championship	Mean Difference (95% CI)	*p* Value	Effect Size
Total Distance (m)	7808 ± 1234 *	8172 ± 1003	−325 (−422 to −229)	*p* < 0.001	−0.32 (−0.72 to 0.07)
Relative Distance (m·min^−1^)	106 ± 17 *	110 ± 14	−4 (−6 to −3)	*p* < 0.001	−0.26 (−0.65 to 0.14)
High-Speed Distance (m)	1215 ± 369	1253 ± 258	−32 (−67 to 4)	*p* = 0.080	−0.12 (−0.51 to 0.27)
Sprint Distance (m)	362 ± 127 *	406 ± 86	−40 (−50 to −29)	*p* < 0.001	−0.41 (−0.80 to −0.01)
Number of Sprints (n)	25 ± 8 *	36 ± 9	−11 (−12 to −11)	*p* < 0.001	−1.29 (−1.71 to 0.85)
Length of Sprint (m)	15 ± 3 *	14 ± 3	1 (1 to 1)	*p* < 0.001	0.33 (−0.06 to 0.73)
Peak Speed (km·h^−1^)	30.0 ± 1.7 *	31.3 ± 1.2	−1.2 (−1.4 to −1.1)	*p* < 0.001	−0.88 (−1.29 to −0.47)

CI = Confidence Interval, Diff = Mean difference, ES = Effect size, * Different (*p* < 0.05) from Championship.

**Table 2:** The total Championship and League running values per player per position are reported. Data are presented as mean ± SD, mean difference (95% CI), and effect size.

		Full Backs	Half Backs	Midfield	Half Forwards	Full Forwards
Total Distance (m)	Championship	7153 ± 357	9150 ± 400 ^a^	9138 ± 512 ^a^	8293 ± 639 ^abc^	7077 ± 380 ^bcd^
	League	6939 ± 713 *	8462 ± 986 *^a^	9141 ± 932 ^a^	8128 ± 934 *^ac^	6516 ± 747 *^bcd^
	Diff (95% CI)	−214 (−15 to −413)	−688 (−865 to −511)	3 (−272 to 278)	−166 (−352 to 21)	−561 (−786 to −337)
	ES	−0.38	−0.91	0.01	−0.21	−0.95
Relative Distance (m·min^−1^)	Championship	101 ± 9	125 ± 5 ^a^	123 ± 7 ^a^	113 ± 9 ^abc^	98 ± 9 ^bcd^
	League	99 ± 14	113 ± 14 *^a^	124 ± 13 ^a^	105 ± 14 *^c^	98 ± 17 ^bc^
	Diff (95% CI)	−2 (−4 to 1)	−12 (−15 to −9)	0 (−4 to 4)	−8 (−11 to −5)	−1 (−3 to 2)
	ES	−0.17	−1.14	0.10	−0.68	0.00
High-Speed Distance (m)	Championship	1030 ± 215	1510 ± 150 ^a^	1435 ± 132 ^a^	1375 ± 175 ^a^	1125 ± 154 ^bcd^
	League	1025 ± 398	1341 ± 314 *^a^	1551 ± 316 *^a^	1259 ± 333 *	1141 ± 279 ^c^
	Diff (95% CI)	−5 (−69 to 59)	−169 (-246 to −93)	115 (14 to −217)	−117 (−197 to −36)	17 (−53 to 87)
	ES	−0.02	−0.69	0.48	−0.44	0.07
Sprint Distance (m)	Championship	374 ± 84	411 ± 83	383 ± 69	440 ± 68	425 ± 98
	League	318 ± 150 *	360 ± 117 *	400 ± 103	334 ± 98 *	421 ± 112 ^a^
	Diff (95% CI)	−56 (−75 to −37)	−50 (−72 to −28)	17 (−13 to 47)	−106 (−130 to −82)	−4 (−25 to 17)
	ES	−0.46	−0.50	0.19	−1.26	−0.04
Number of Sprints (n)	Championship	33 ± 8	44 ± 7 ^a^	36 ± 9	36 ± 8 ^b^	31 ± 8 ^b^
	League	24 ± 12 *	24 ± 8 *	26 ± 5 *	23 ± 7 *	27 ± 6 *
	Diff (95% CI)	−9 (−11 to −8)	−20 (−22 to −18)	−10 (−12 to −8)	−13 (−15 to −11)	−4 (−6 to −3)
	ES	−0.88	−2.66	−1.37	−1.73	−0.57
Length of Sprint (m)	Championship	13 ± 3	14 ± 2	13 ± 3	14 ± 3	16 ± 3 ^abc^
	League	14 ± 4 *	15 ± 3 *	15 ± 2 *	15 ± 2	16 ± 3
	Diff (95% CI)	1 (−1 to 0)	2 (1 to 2)	2 (1 to 3)	0 (0 to 1)	1 (−1 to 0)
	ES	0.28	0.39	0.78	0.39	0.00
Peak Speed (Km·h^−1^)	Championship	31.5 ± 1.2	31.0 ± 1.0	30.6 ± 1.2	31.2 ± 1.0	31.9 ± 1.0 ^bc^
	League	29.5 ± 2.0 *	29.8 ± 1.2 *	30.2 ± 1.8 *	29.6 ± 1.4 *	30.9 ± 1.6 *^a^
	Diff (95% CI)	−2.0 (−2.2 to −1.7)	−1.2 (−1.5 to −0.9)	−0.4 (−0.8 to 0.0)	−1.6 (−1.9 to −3)	−1.0 (−1.3 to −0.8)
	ES	−1.21	−1.09	−0.26	−1.32	−0.75

CI = Confidence Interval, Diff = Mean difference, ES = Effect size, * Different (*p* < 0.05) from Championship, ^a^ Different (*p* < 0.05) from full backs, ^b^ Different (*p* < 0.05) from half backs, ^c^ Different (*p* < 0.05) from midfielders, ^d^ Different (*p* < 0.05) from half forwards.

## Data Availability

The data presented in this study are available on request from the corresponding author.
